# Demographic inference from multiple whole genomes using a particle filter for continuous Markov jump processes

**DOI:** 10.1371/journal.pone.0247647

**Published:** 2021-03-02

**Authors:** Donna Henderson, Sha (Joe) Zhu, Christopher B. Cole, Gerton Lunter

**Affiliations:** 1 Wellcome Centre for Human Genetics, Oxford, United Kingdom; 2 Big Data Institute, Oxford, United Kingdom; 3 MRC Weatherall Institute of Molecular Medicine, John Radcliffe Hospital, Headington, Oxford, United Kingdom; 4 Department of Epidemiology, University Medical Center Groningen, University of Groningen, Groningen, The Netherlands; Universidad Rey Juan Carlos, SPAIN

## Abstract

Demographic events shape a population’s genetic diversity, a process described by the coalescent-with-recombination model that relates demography and genetics by an unobserved sequence of genealogies along the genome. As the space of genealogies over genomes is large and complex, inference under this model is challenging. Formulating the coalescent-with-recombination model as a continuous-time and -space Markov jump process, we develop a particle filter for such processes, and use waypoints that under appropriate conditions allow the problem to be reduced to the discrete-time case. To improve inference, we generalise the Auxiliary Particle Filter for discrete-time models, and use Variational Bayes to model the uncertainty in parameter estimates for rare events, avoiding biases seen with Expectation Maximization. Using real and simulated genomes, we show that past population sizes can be accurately inferred over a larger range of epochs than was previously possible, opening the possibility of jointly analyzing multiple genomes under complex demographic models. Code is available at https://github.com/luntergroup/smcsmc.

## Introduction

The demographic history of a species has a profound impact on its genetic diversity. Changes in population size, migration and admixture events, and population splits and mergers, shape the genealogies describing how individuals in a population are related, which in turn shape the pattern and frequency of observed genetic variants in extant genomes. By modeling this process and integrating out the unobserved genealogies, it is possible to infer the population’s demographic history from the observed variants. However, in practice this is challenging, as individual mutations provide limited information about tree topologies and branch lengths. If many mutations were available to infer these genealogies this would not be problematic, but the expected number of observed mutations increases only logarithmically with the number of observed genomes, and recombination causes genealogies to change along the genome at a rate proportional to the mutation rate. As a result there is considerable uncertainty about the genealogies underlying a sample of genomes, and because the space of genealogies across the genome is vast, integrating out this latent variable is hard.

A number of approaches have been proposed to tackle this problem [reviewed in 1]. A common approximation is to treat recombination events as known and assume unlinked loci, either by treating each mutation as independent [2–7], or by first identifying tracts of genetic material unbroken by recombination [8–12]. To account for recombination while retaining power to infer earlier demographic events, it is necessary to model the genealogy directly. ARGWeaver [13] uses Markov chain Monte Carlo (MCMC) for inference, but does not allow the use of a complex demographic model, and since mutations are only weakly informative about genealogies this leaves the inferred trees biased towards the prior model and less suitable for inferring demography. Restricting itself to single diploid genomes, the Pairwise Sequentially Markovian Coalescent (PSMC) model [14] uses an elegant and efficient inference method, but with limited power to detect recent changes in population size or complex demographic events. Several other approaches exist that improve on PSMC in various ways [15–18], but they remain limited particularly in their ability to infer migration.

We here focus on the general problem of inferring demography from several whole-genome sequences, which is informative about demographic events in all but the most recent epochs [13, 14, 16]. A promising approach which so far has not been applied to this problem is to use a particle filter. Particle filters have many desireable properties [19–22], and applications to a range of problems in computational biology have started to appear [23–26]. Like MCMC methods, particle filters converge to the exact solution in the limit of infinite computational resources, are computationally efficient by focusing on realisations that are supported by the data, do not require the underlying model to be approximated, and generate explicit samples from the posterior distribution of the latent variable. Unlike MCMC, particle filters do not operate on complete realisations of the model, but construct samples sequentially, which is helpful since full genealogies over genomes are cumbersome to deal with.

To use particle filters, we use a formulation of the coalescent model in which the state is a genealogical tree at a particular genome locus, which “evolves” sequentially along the genome, rather than in evolutionary time. To avoid confusion, in this paper “time” by itself refers to the variable along which the model evolves, while evolutionary (coalescent, recombination) time refers to an actual time in the past on a genealogical tree.

Originally, particle filters were introduced for models with discrete time evolution and with either discrete or continuous state variables [19, 27]. In this paper, the latent variable is a piecewise constant sequence of genealogical trees along the genome, with trees changing only after recombination events that, in mammals, occur once every several hundred nucleotides. The observations of the model are genetic variants, which are similarly sparse. Realizations of the discrete-time model of this process (where “time” is the genome locus) are therefore stationary (remain in the same state) and silent (do not produce an observation) at most transitions, leading to inefficient algorithms. Instead, it seems natural to model the system as a Markov jump process (or purely discontinuous Markov process, [28]), a continuous-time stochastic process with as realisations piecewise constant functions x:[1,L]↦T, where T is the state space of the Markov process (the space of genealogical trees over a given number of genomes) and *L* the length over which observations are made (here the genome size).

Particle filters have been generalised to continuous-time diffusions [29–31], as well as to Markov jump processes on discrete state spaces [32, 33], and hybrids of the two [34, 35], as well as to piecewise deterministic processes [36]; for a general treatment see [37, 38]. Here we focus on Markov jump processes that are continuous in both time and state space; to our knowledge the method has not been extended to this case. The algorithm we propose relies on Radon-Nikodym derivatives [see e.g. 31], and we establish criteria for choosing a finite set of “waypoints” that makes it possible to reduce the problem to the discrete-time case, while ensuring that particle degeneracy remains under control.

Although the algorithm generally works well, we found that for the CwR model we obtain biased inferences for some parameters. For example, coalescent rates for recent epochs are associated with tree nodes that persist across long genomic segments (the model exhibits “long forgetting times”), because their short descendant branches attract few recombinations. They have few informative mutations as well, and collecting these mutations therefore require long lags in the fixed-lag smoothing procedure, in turn resulting in increased particle degeneracy [39]. For discrete-time models the Auxiliary Particle Filter [40] addresses a related problem by “guiding” the particle filter towards states that are likely to be relevant in future iterations, using an approximate likelihood that depends on data one step ahead. This approach does not work well for some continuous-time models, including ours, that have no single preferred time scale. Instead we introduce an algorithm that shapes the resampling process by an approximate “lookahead likelihood” that can depend on data at arbitrary distances ahead. Using simulations we show that this substantially reduces the bias.

The particle filter generates samples from the posterior distribution of the latent variable, here the sequence of genealogies along the genome, and we infer the model parameters from this sample. One strategy is to use stochastic expectation-maximization [SEM; 41]. However, such approaches yield point estimates, ignoring any uncertainty in the inferred parameters. Combined with the bias due to self-normalized importance sampling which cause particle filters to under-sample low-rate events, this result in a non-zero probability of inferring zero event rates, which are fixed points of any SEM procedure. In principle this can be avoided by using an appropriate prior on the rate parameters. To implement this we use Variational Bayes to estimate an approximate joint posterior distribution over parameters and latent variables, partially accounting for the uncertainty in the inferred parameters, as well as providing way to explicitly include a prior. In this way zero-rate estimates are avoided, and more generally we show that this approach further reduces the bias in parameter estimates.

Applying these ideas to the coalescent-with-recombination (CwR) model, we find that the combination of lookahead filter and Variational Bayes inference enables us to analyze four diploid human genomes simultaneously, and infer demographic parameters across epochs spanning more than 3 orders of magnitude, without making model approximations beyond passing to a continuous-locus model.

The remainder of the paper is structured as follows. We first introduce the particle filter, generalise it to continuous-time and -space Markov jump processes, describe how to choose waypoints, introduce the lookahead filter, and describe the Variational Bayes procedure for parameter inference. In the results section we first introduce the continuous-locus CwR process, then discuss the lookahead likelihood, choice of waypoints and parameter inference for this model, before applying the model to simulated data, and finally show the results of analyzing sets of four diploid genomes of individuals from three human populations. A discussion concludes the paper.

## Methods

### The sequential coalescent with recombination model

The coalescent-with-recombination (CwR) process, and the graph structures that are the realisations of the process, was first described by Hudson [42], and was given an elegant mathematical description by Griffiths [43], who named the resulting structure the Ancestral Recombination Graph (ARG). Like the coalescent process, these models run backwards in evolutionary time and consider the entire sequence at once, making it difficult to use them for inference on whole genomes. The first model of the CwR process that evolves sequentially rather than in the evolutionary time direction was introduced by Wiuf and Hein [44], opening up the possibility of inference over very long sequences. Like Griffiths’ process, the Wiuf-Hein algorithm operates on an ARG-like graph, but it is more efficient as it does not include many of the non-observable recombination events included in Griffiths’ process. The Sequential Coalescent with Recombination Model (SCRM) [45] further improved efficiency by modifying Wiuf and Hein’s algorithm to operate on a local genealogy rather than an ARG-like structure. Besides the “local” tree over the observed samples, this genealogy includes branches to non-contemporaneous tips that correspond to recombination events encountered earlier in the sequence. Recombinations on these “non-local” branches can be postponed until they affect observed sequences, and can sometimes be ignored altogether, leading to further efficiency gains while the resulting sample still follows the exact CwR process. An even more efficient but approximate algorithm is obtained by culling some non-local branches. In the extreme case of culling *all* non-local branches the SCRM approximation is equivalent to the SMC’ model [46, 47]. With a suitable definition of “current state” (i.e., the local tree including all non-local branches) these are all Markov processes, and can all be used in the Markov jump particle filter; here we use the SCRM model with tunable accuracy as implemented in [45].

The state space T of the Markov process is the set of all possible genealogies at a given locus. The probability measure of a complete realisation *x* can be written as

$$\begin{array}{c} \pi_{x}\left(x\right)=\text{exp}\;\{\;-\;\int \;B\left(x_{s}\right)\rho \left(s\right)\text{d}s\;\}\;[\prod_{j=1}^{\left|x\right|}\text{exp}\;\{\;-\;\int_{\nu_{j}}^{\tau_{j}}\;b_{u}\left(x_{s_{j}}\right)C\left(u\right)\text{d}u\;\}\;\rho \left(s_{j}\right)C\left(\tau_{j}\right)]\;{\left(\text{d}s\right)}^{\left|x\right|}{\left(\text{d}u\right)}^{2|x|}. \end{array}$$ (1)

Here *x* is the sequence of genealogies along the genome; |*x*| is the number of recombinations that occurred on *x*; *b*_*u*_(*x*_*s*_) is the number of branches in the genealogy at locus *s* at evolutionary time *u*; $B\left(x_{s}\right)=\int_{u=0}^{\text{root}(x_{s})}b_{u}\left(x_{s}\right)\text{d}u$ is the total branch length of *x*_*s*_; *ρ*(*s*) is the recombination rate per nucleotide and per generation at locus *s*, so that *ρ*(*s*)*B*(*x*_*s*_) is the exit rate of the Markov process in state *x*_*s*_; (*s*_*j*_, *ν*_*j*_) is the locus and recombination time of the *j*th recombination event; *τ*_*j*_ > *ν*_*j*_ is the coalescence time of the corresponding coalescence event; and *C*(*u*) = 1/2*N*_*e*_(*u*) is the coalescence rate in generation *u*. See Appendix (“The sequential coalescent with recombination process”) for more details. The distribution *π*_*x*_(*x*) has a density with respect to the Lebesgue measure (d*s*)^|*x*|^(d*u*)^2|*x*|^, because each of the |*x*| recombination events is associated with a sequence locus, a recombination time, and a coalescent time.

Mutations follow a Poisson process whose rate at *s* depends on the state *x*_*s*_ via *μ*(*s*)*B*(*x*_*s*_) where *μ*(*s*) is the mutation rate at *s* per nucleotide and per generation. Mutations are not observed directly, but their descendants are; a complete observation is represented by a set $y={\left\{\left(s_{j},A_{j}\right)\right\}}_{j=1,\ldots ,|y|}\in Y$ where *s*_*j*_ ∈ [1, *L*) is the locus of mutation *j*, and *A*_*j*_ ∈ {0, 1}^*S*^ are the wildtype (0) and alternative (1) alleles observed in the *S* samples. The conditional probability measure of the observations *y* given a realisation *x* is

$$\begin{array}{c} \pi \left(y|X=x\right)=\frac{1}{|y|!}\text{exp}\{-\int B\left(x_{s}\right)\mu \left(s\right)\text{d}s\}[\prod_{j=1}^{\left|y\right|}P\left(A_{j}|x_{s_{j}},\mu \left(s_{j}\right)\right)]{\left(\text{d}s\right)}^{\left|y\right|} \end{array}$$ (2)

where *P*(*A*|*x*_*s*_, *μ*) is the probability of observing the allelic pattern *A* given a genealogy *x*_*s*_ and a mutation rate *μ* per nucleotide and per generation; this probability is calculated using Felsenstein’s peeling algorithm [48]. Note that *B*(*x*_*s*_)*μ*(*s*) = ∑_*A*≠(0,…,0)_ *P*(*A*|*x*_*s*_, *μ*(*s*)).

### Particle filters

Particle filters methods, also known as Sequential Monte Carlo (SMC) [22], generate samples from complex probability distributions with high-dimensional latent variables. An SMC method uses importance sampling (IS) to approximate a target distribution using weighted random samples (particles) drawn from a tractable distribution. We briefly review the discrete-time case. Suppose that particles {(*x*^(*i*)^, *w*^(*i*)^)}_*i*=1,…,*N*_, approximate a distribution with density *p*(*x*), such that

$$\begin{array}{c} E_{p}\left[f\left(X\right)\right]=\int f\left(x\right)p\left(x\right)\text{d}x\approx \frac{1}{\sum_{i=1}^{N}w^{\left(i\right)}}\sum_{i=1}^{N}w^{\left(i\right)}f\left(x^{\left(i\right)}\right) \end{array}$$ (3)

for any bounded continuous function *f*, where *X* ∼ *p*(*x*)*dx*. Here and in the remainder, we use “approximate” and ≈ to mean that *X*_*N*_ ∼ ∑*w*^(*i*)^ *δ*_*x*^(*i*)^_(*x*) converges in distribution to *X* ∼ *p*(*x*)d*x* and equality holds in (3) as *N* → ∞; and summations without an index are over *N* particles indexed by *i*. Under mild conditions (i.e., *q*(*x*)/*p*(*x*) must exist almost everywhere and be absolutely continuous) we can use IS to obtain particles approximating another distribution *q*(*x*)d*x*:

$$\begin{array}{c} E_{q}\left[f\left(X\right)\right]=\;\int \;f\left(x\right)\frac{q(x)}{p(x)}p\left(x\right)\text{d}x\approx \frac{1}{\sum \;w^{\left(i\right)}}\;\sum w^{\left(i\right)}\frac{q(x^{\left(i\right)})}{p(x^{\left(i\right)})}f\left(x^{\left(i\right)}\right)\approx \frac{1}{\sum \;{\tilde{w}}^{\left(i\right)}}\;\sum {\tilde{w}}^{\left(i\right)}f\left(x^{\left(i\right)}\right), \end{array}$$

where ${\tilde{w}}^{\left(i\right)}:=w^{\left(i\right)}q\left(x^{\left(i\right)}\right)/p\left(x^{\left(i\right)}\right)$, and the last step holds because $\sum {\tilde{w}}^{\left(i\right)}/\sum w^{\left(i\right)}\approx E_{p}\left[q/p\right]=E_{q}\left[1\right]=1$. This shows that $\left\{\left(x^{\left(i\right)},{\tilde{w}}^{\left(i\right)}\right)\right\}$ approximate *q*(*x*)d*x*. The normalisation ensures that any constant factor in *w*^(*i*)^ drops out, so that it is sufficient to know the ratio *q*(*x*)/*p*(*x*) up to a constant. A particle filter builds the desired distribution sequentially, making it suited to hidden Markov models, for which the joint distribution of latent variables *X* and observations *Y* has the form

$$\begin{array}{c} P(X=x_{1\cdot \;\cdot s})=p\left(x_{1}\right)p\left(x_{2}|x_{1}\right)\cdots p\left(x_{s}|x_{s-1}\right) \end{array}$$ (4)

$$\begin{array}{c} P(Y=y_{1\cdot \;\cdot s}|X=x_{1\cdot \;\cdot s})=g\left(y_{1}|x_{1}\right)\cdots g\left(y_{s}|x_{s}\right) \end{array}$$ (5)

Here 1··s denotes the set {1, 2, …, *s*}, and $x=x_{1\cdot \;\cdot s}=\left(x_{1},x_{2},\ldots ,x_{s}\right)$ and $y=y_{1\cdot \;\cdot s}$ are vectors. Let {(*x*^(*i*)^, *w*^(*i*)^)} be particles approximating the target distribution $P(X_{1\cdot \;\cdot s}=x_{1\cdot \;\cdot s}|Y_{1\cdot \;\cdot s}=y_{1\cdot \;\cdot s})$, which for brevity we write as $P(x_{1\cdot \;\cdot s}|y_{1\cdot \;\cdot s})$. If ${\tilde{x}}^{\left(i\right)}$ is the vector obtained by extending *x*^(*i*)^ with a sample from $P(x_{s+1}|x_{s}^{\left(i\right)})$, then from (4) and (5) it follows that $\left\{\left({\tilde{x}}^{\left(i\right)},w^{\left(i\right)}\right)\right\}$ approximate $P(x_{1\cdot \;\cdot s+1}|y_{1\cdot \;\cdot s})\propto$ $P(x_{1\cdot \;\cdot s+1},y_{1\cdot \;\cdot s})$. Now, $P(x_{1\cdot \;\cdot s+1}|y_{1\cdot \;\cdot s+1})\propto$ $P(x_{1\cdot \;\cdot s+1},y_{1\cdot \;\cdot s+1})=$ $P\left(x_{1\cdot \;\cdot s+1},y_{1\cdot \;\cdot s}\right)g\left(y_{s+1}|x_{s+1}\right)$, so that using IS and setting

$$\begin{array}{c} {\tilde{w}}^{\left(i\right)}=w^{\left(i\right)}g\left(y_{s+1}|{\tilde{x}}_{s+1}^{\left(i\right)}\right) \end{array}$$ (6)

we obtain particles $\left\{\left({\tilde{x}}^{\left(i\right)},{\tilde{w}}^{\left(i\right)}\right)\right\}$ that approximate $P(x_{1\cdot \;\cdot s+1}|y_{1\cdot \;\cdot s+1})$. This shows how to sequentially construct particles that approximate the target distribution $P(x_{1\cdot \;\cdot L}|y_{1\cdot \;\cdot L})$. Instead of sampling from $p(x_{s+1}|x_{s}^{\left(i\right)})$, any proposal distribution $q(x_{s+1}|x_{s}^{\left(i\right)},y_{1\cdot \;\cdot L})$ (subject to conditions) can be used, which is advantageous if *q* is easier to sample from, is closer to the target distribution, or has heavier tails than *p*. Again, IS accounts for the change in sampling distribution, resulting in

$$\begin{array}{c} {\tilde{w}}^{\left(i\right)}=w^{\left(i\right)}g\left(y_{s+1}|{\tilde{x}}_{s+1}^{\left(i\right)}\right)\frac{p({\tilde{x}}_{s+1}^{\left(i\right)}|x_{s}^{\left(i\right)})}{q({\tilde{x}}_{s+1}^{\left(i\right)}|x_{s}^{\left(i\right)})}. \end{array}$$ (7)

For now we will choose *q* to be independent of *y*. Because samples from *q* do not follow the desired target *P*(*x*|*y*), the fraction of particles close to the target’s mode diminishes exponentially at each iteration until (3) fails altogether. To address this, we occasionally draw samples from the approximating distribution itself, assigning each resampled particle weight 1/*N*—interestingly, if we interpret fitness as (proportional to) the likelihood $g(y_{s+1}|{\tilde{x}}_{s+1}^{\left(i\right)})$, this is the same process that is used in the Wright-Fisher model with selection to describe how fitness differences shape an evolving constant-size population [49]. Doing this tends to remove particles that have drifted from the mode of the target and have low weight, and duplicates particles with large weights, while (3) remains valid. Although resampling substantially decreases the future variance of (3), it increases the variance at the current iteration. To avoid increasing this variance unnecessarily, resampling is performed only when the estimated sample size, defined as *ESS* = (∑*w*^(*i*)^)^2^/∑(*w*^(*i*)^)^2^, drops below a threshold, e.g. *N*/2. In addition, we use systematic resampling to minimize the variance that is introduced when resampling is performed [50]. This leads to Algorithm 1 [19].

Note that the algorithm can be seen as a recipe to transform a sample from *P*(*X*) to a sample from *P*(*X*)*P*(*Y*|*X*)/*P*(*Y*) = *P*(*X*|*Y*), that is, an application of Bayes’ theorem. Following this interpretation we will refer to *P*(*X*) as the prior distribution, and *P*(*X*|*Y*) as the posterior.

The algorithm generates an approximation to $P(x_{1\cdot \;\cdot s}|y_{1\cdot \;\cdot s})$ rather than $P(x_{s}|y_{1\cdot \;\cdot s})$, but we follow [22] in calling it a particle filter algorithm instead of a smoothing algorithm (although our use of fixed-lag distributions for parameter estimation is a partial smoothing operation).

The marginal likelihood can be estimated (although with high variance, see [51]) by setting the weights to $N^{-1}\sum_{i}w_{s}^{\left(i\right)}$ rather than *N*^−1^ when particles are resampled. This makes the weights asymptotically normalized, so that (3) becomes *E*_*P*(*X*,*Y*=*y*)_[*f*] ≈ ∑_*i*_ *w*^(*i*)^ *f*(*x*^(*i*)^), and $P\left(Y=y\right)=\int P\left(x,y\right)\text{d}x=E_{P(X,Y=y)}\left[1\right]\approx \sum_{i}w_{L}^{\left(i\right)}$.

**Algorithm 1** Particle filter

**Input**: $y_{1\cdot \;\cdot L}$

**Output**: Particles $\left\{\left(x_{1\cdot \;\cdot L}^{\left(i\right)},w_{L}^{\left(i\right)}\right)\right\}$ approximating $P(x_{1\cdot \;\cdot L}|y_{1\cdot \;\cdot L})$

 $w_{0}^{\left(i\right)}\leftarrow 1/N$, $x_{0}^{\left(i\right)}\leftarrow \emptyset \left(i=1,\ldots ,N\right)$

 For *s* from 0 to *L* − 1

  **Loop invariant**: $\left\{\left(x_{1\cdot \;\cdot s}^{\left(i\right)},w_{s}^{\left(i\right)}\right)\right\}∼p\left(x_{1\cdot \;\cdot s}|y_{1\cdot \;\cdot s}\right)$

  If *ESS* < *N*/2:

   Resample, with replacement, $\left\{x_{1\cdot \;\cdot s}^{\left(i\right)}\right\}$ proportional to $\left\{w_{s}^{\left(i\right)}\right\}$

   $w_{s}^{\left(i\right)}\leftarrow N^{-1}\left(i=1,\ldots ,N\right)$

  For *i* from 1 to *N*:

   Sample $x_{s+1}^{\left(i\right)}∼q\left(x_{s+1}|x_{s}^{\left(i\right)}\right)$

   $w_{s+1}^{\left(i\right)}\leftarrow w_{s}^{\left(i\right)}\frac{p(x_{s+1}^{\left(i\right)}|x_{s}^{\left(i\right)})}{q(x_{s+1}^{\left(i\right)}|x_{s}^{\left(i\right)})}g\left(y_{s+1}|x_{s+1}^{\left(i\right)}\right)$.

### Continuous-time and -space Markov jump processes

For the hidden process we now consider Markov jump processes, which have as realisations piecewise constant functions x:[1,L)↦T where T is the state space of the Markov process. Recall that in the model we consider, T is the space of rooted genealogical trees with branch lengths. Let $\left(X,F_{x},\pi_{x}\right)$ be a probability space, where $X=T^{\left[1,L\right)}$ is the space of possible realisations of the hidden stochastic process *X* = {*X*_*s*_}_*s* ∈ [1, *L*)_, $F_{x}\subset P\left(X\right)$ is the *σ*-algebra of events, and *π*_*x*_(*X*) is the probability measure on X induced by the stochastic process *X*. See the Appendix (“Conditional distributions and the Markov property”) for some remarks on how to define a Markov model when the phase space T is uncountable.

The complete model is defined by specifying the observation process. We consider models where observations *Y* are generated by a Poisson process whose intensity at time (i.e. locus) *s* depends on *X*_*s*_ [a Cox process, see e.g. 52]. The space of observations Y consists of finite subsets of [1, *L*) × *M*, where *M* is a discrete set of potential events, each of which may occur at some *s* ∈ [1, *L*). For a full observation $y=(\left({\tilde{s}}_{1},m_{1}\right),\ldots ,\left({\tilde{s}}_{k},m_{k}\right))\in Y$ we write |*y*| ≔ *k* for the number of events in *y*. Writing λ(*y*) for the Lebesgue measure (d*s*)^|*y*|^, the emission distribution *π*(*Y*|*X* = *x*) has a density *r*(*y*|*x*) relative to λ(*y*). For Cox processes this density has the form

$$\begin{array}{c} r\left(y|x\right)=\frac{1}{|y|!}\text{exp}(-\int_{s=1}^{L}r\left(x_{s}\right))\prod_{i=1}^{\left|y\right|}r\left({\tilde{s}}_{i},m_{i}|x_{{\tilde{s}}_{i}}\right) \end{array}$$ (8)

where *r*(*s*, *m*|*x*_*s*_) is the rate at which event *m* occurs at time *s* conditional on *X*_*s*_ = *x*_*s*_ and

$$\begin{array}{c} r\left(x_{s}\right):=\sum_{m\in M}r\left(s,m|x_{s}\right)\text{d}s \end{array}$$ (9)

is the intensity of the emission Poisson process at *s* conditional on *X*_*s*_ = *x*_*s*_. The probability space for the joint process is (X×Y,F,π), and the posterior distribution of interest is *π* conditioned on an observation y∈Y, written as *π*(*X*|*Y* = *y*).

The *absence* of events in an interval *s* ∈ [*a*, *b*) is also informative about the latent variable through the exponential factor in (8). In practice however, not all intervals may have been observed, so that events may or may not have occurred in these intervals. Assuming that the “observation process” is independent of the Markov jump process *X*, such unobserved intervals can simply be left out of integral (8).

Some more notation is needed to describe the Markov jump process version of algorithm 1. As above *π*_*x*_ denotes the prior distribution of the latent variable *X*, and *ξ*_*x*_ denotes the proposal distribution, both Markov processes on X, playing the role of *p*(*x*) and *q*(*x*) in the discrete case. We write a:b for the interval [a,b)⊂R, and *α*^*a*: *b*^ for the restriction of a measure or function *α* to a:b; similarly *y*_*a*:*b*_ ≔ *y*∩([*a*, *b*) × *M*) and *X*_*a*:*b*_ ≔ {*X*_*s*_}_*s*∈[*a*,*b*)_. The particle filter algorithm uses the notation (d*α*/d*β*)(*x*) for distributions *α* and *β* to denote their Radon-Nikodym derivative: the ratio of their density functions with respect to a common reference measure, evaluated at *x*. To simplify notation we write the Radon-Nikodym derivative of two conditional distributions α(X|G) and β(X|G) at *x* as (dα/dβ)(x|G), and we also do not explicitly restrict distributions to their appropriate intervals when this is clear from the context, so that we write for example $\left(\text{d}\pi /\text{d}\lambda \right)\left(y_{s_{j}:s_{j+1}}|X_{s_{j}:s_{j+1}}=x\right)$ instead of $\left(\text{d}\pi^{s_{j}:s_{j+1}}\left(Y|X_{s_{j}:s_{j+1}}=x\right)/\text{d}\lambda^{s_{j}:s_{j+1}}\right)\left(y_{s_{j}:s_{j+1}}\right)$. With this notation we can formulate Algorithm 2.

**Algorithm 2** Particle filter for Markov jump processes

**Input**: $y_{1:L}\in Y$; waypoints 1 = *s*_0_ < *s*_1_ < … < *s*_*K*_ = *L*.

**Output**: Particles $\left\{\left(x_{1:L}^{\left(i\right)},w_{L}^{\left(i\right)}\right)\right\}$ approximating the posterior distribution *π*(*X*|*Y* = *y*_1:*L*_)

  $w_{1}^{\left(i\right)}\leftarrow N^{-1}$, $x_{1:1}^{\left(i\right)}\leftarrow \emptyset$ (*i* = 1, …, *N*)

 For *j* from 0 to *K* − 1

  **Loop invariant**: $\left\{\left(x_{1:s_{j}}^{\left(i\right)},w_{s_{j}}^{\left(i\right)}\right)\right\}\approx \pi \left(X_{1:s_{j}}|Y_{1:s_{j}}=y_{1:s_{j}}\right)$

  If $ESS(\left\{w_{s_{j}}^{\left(i\right)}\right\})<N/2$:

   Resample $\left\{x_{1:s_{j}}^{\left(i\right)}\right\}$ with probabilities proportional to $\left\{w_{s_{j}}^{\left(i\right)}\right\}$

   $w_{s_{j}}^{\left(i\right)}\leftarrow N^{-1}$ (*i* = 1, …, *N*)

  For *i* from 1 to *N*:

   Sample $x_{s_{j}:s_{j+1}}^{\left(i\right)}∼\xi_{x}\left(X_{s_{j}:s_{j+1}}|X_{s_{j}}=x_{s_{j}}^{\left(i\right)}\right)$

   $w_{s_{j+1}}^{\left(i\right)}\leftarrow w_{s_{j}}^{\left(i\right)}\frac{\text{d}\pi_{x}}{\text{d}\xi_{x}}\left(x_{s_{j}:s_{j+1}}^{\left(i\right)}|X_{s_{j}}=x_{s_{j}}^{\left(i\right)}\right)\frac{\text{d}\pi }{\text{d}\lambda }\left(y_{s_{j}:s_{j+1}}|X_{s_{j}:s_{j+1}}=x_{s_{j}:s_{j+1}}^{\left(i\right)}\right)$.

The choice of waypoints *s*_1_, …, *s*_*K*_ is discussed below; in particular they need not be the same as the event loci ${\tilde{s}}_{1},\ldots ,{\tilde{s}}_{\left|y\right|}$ of the observation *y*. Note that there is no initialization step; instead, initially $x_{1:1}^{\left(i\right)}=\emptyset$, and the first sample will be drawn from *ξ* conditioned on an empty set, i.e. the unconditional distribution. The loop invariant holds when *j* = 0 since $1\;:\;s_{0}=\emptyset$. As with Algorithm 1 it is possible to estimate the likelihood density *π*_*θ*_(*y*_1:*L*_) by replacing the factors *N*^−1^ with $N^{-1}\sum_{i}w_{s_{j}}^{\left(i\right)}$; then the likelihood density w.r.t. λ(d*y*) = (d*s*)^|*y*|^ is approximated by $\sum_{i}w_{L}^{\left(i\right)}$.

Note that by the nature of Markov jump processes, particles that start with identical latent variables have a positive probability of remaining identical after a finite time. Combined with resampling, this causes a considerable number of particles to have one or more identical siblings. For computational efficiency we represent such particles once, and keep track of their multiplicity *k*. When evolving a particle with multiplicity *k* > 1, we increase the exit rate *k*-fold, and when an event occurs one particle is spawned off while the remaining *k* − 1 continue unchanged.

### Using lookahead to improve the particle filter

At the *j*th iteration, Algorithm 2 uses data up to waypoint *s*_*j*_ to build particles approximating $\pi (X_{1:s_{j}}|Y_{1:s_{j}}=y_{1:s_{j}})$. This is reasonable as $\pi (X_{1:s_{j}}|y_{1:s_{j}})$ is independent of data beyond *s*_*j*_. However, not all particles are equally important for approximating subsequent posteriors, which suggests to emphasise particles that will be relevant in future at the expense of those relevant only to $\pi (X_{1:s_{j}}|y_{1:s_{j}})$. This echoes the justification of resampling: although resampling increases the variance of the approximation to the current partial posterior, the variance at subsequent iterations by increasing the number of particles that are likely to contribute to future distributions. For discrete-time models *p*(*X*_1:*n*_|*y*_1:*n*_), the Auxiliary Particle Filter (APF) [40] implements this intuition by targeting a resampling distribution [53], which includes a “lookahead” factor $\tilde{p}\left(y_{i+1}|x_{i}\right)$ approximating the probability of observing data *y*_*i*+1_ given the current state *x*_*i*_. Importance sampling is used to keep track of the desired distribution *p*(*X*_1:*i*_|*y*_1:*i*_).

In the continuous-time context it is natural to look an arbitrary distance ahead. Similar to APF, the lookahead distribution can be conditioned on the current state only, and must be an approximation of the true distribution. It should be heavy-tailed with respect to the true distribution to ensure that the variance of the estimator remains finite [22], which implies that the distribution should not depend on data too far beyond *s*; what is “too far” depends on how well the lookahead distribution approximates the true distribution.

The lookahead distribution is only evaluated on a fixed observation *y*, and is used to quantify the plausibility of a current state $x_{s}^{\left(i\right)}$, rather than to define a distribution over *y*. For this reason we call it a lookahead *likelihood*. In fact, for correctness of the algorithm it is not necessary that this likelihood derives from a probability distribution. We define the lookahead likelihood as a family of functions $h^{s}\left(y_{s:L}|x_{s}\right):Y_{s:L}\times T\to R$, and an associated family of unnormalized distributions ${\tilde{\pi }}^{s}\left(x_{1:s},y_{1:L}\right)=\pi^{1:s}\left(x_{1:s},y_{1:s}\right)h^{s}\left(y_{s:L}|x_{s}\right)\lambda^{s:L}\left(y_{s:L}\right)$ on $X_{1:s}\times Y$. The functions *h*^*s*^ can be chosen arbitrarily, except that *h*^*s*^(⋅, *x*_*s*_)λ^*s*:*L*^ must be absolutely continuous w.r.t. *π*^*s*:*L*^(⋅|*X*_*s*_ = *x*_*s*_) to ensure that importance sampling is justified. The lookahead Algorithm 3 keeps track of two sets of weights, which together with a single set of samples form two sets of particles that approximate the resampling and target distributions.

**Algorithm 3** Markov-jump particle filter with lookahead

**Input**: $y_{1:L}\in Y$; waypoints 1 = *s*_0_ < *s*_1_ < … < *s*_*K*_ = *L*.

**Output**: Particles $\left\{\left(x_{1:L}^{\left(i\right)},w_{L}^{\left(i\right)}\right)\right\}$ approximating *π*(*X*|*Y*_1:*L*_ = *y*_1:*L*_)

 $w_{1}^{\left(i\right)}\leftarrow 1/N$, $v_{1}^{\left(i\right)}\leftarrow 1/N$, $x_{1:1}^{\left(i\right)}\leftarrow \emptyset$ (*i* = 1, …, *N*)

 For *j* from 0 to *K* − 1

  **Loop invariant**: $\left\{\left(x_{1:s_{j}}^{\left(i\right)},w_{s_{j}}^{\left(i\right)}\right)\right\}\approx \pi^{1:s_{j}}\left(X_{1:s_{j}}|Y_{1:s_{j}}=y_{1:s_{j}}\right)$

  **Loop invariant**: $\left\{\left(x_{1:s_{j}}^{\left(i\right)},v_{s_{j}}^{\left(i\right)}\right)\right\}\approx {\tilde{\pi }}^{s_{j}}\left(X_{1:s_{j}}|Y=y_{1:L}\right)$

  If $ESS(\left\{v_{s_{j}}^{\left(i\right)}\right\})<N/2$:

   Resample $\left\{x_{1:s_{j}}^{\left(i\right)}\right\}$ with probabilities proportional to $\left\{v_{s_{j}}^{\left(i\right)}\right\}$

   $w_{s_{j}}^{\left(i\right)}\leftarrow N^{-1}w_{s_{j}}^{\left(i\right)}/v_{s_{j}}^{\left(i\right)}$ (*i* = 1, …, *N*)

   $v_{s_{j}}^{\left(i\right)}\leftarrow N^{-1}$ (*i* = 1, …, *N*)

  For *i* from 1 to *N*:

   Sample $x_{s_{j}:s_{j+1}}^{\left(i\right)}∼\xi_{x}\left(X_{s_{j}:s_{j+1}}|X_{s_{j}}=x_{s_{j}}^{\left(i\right)}\right)$

   $w_{s_{j+1}}^{\left(i\right)}\leftarrow w_{s_{j}}^{\left(i\right)}\frac{\text{d}\pi_{x}}{\text{d}\xi_{x}}\left(x_{s_{j}:s_{j+1}}^{\left(i\right)}|X_{s_{j}}=x_{s_{j}}^{\left(i\right)}\right)\frac{\text{d}\pi }{\text{d}\lambda }\left(y_{s_{j}:s_{j+1}}|X_{s_{j}:s_{j+1}}=x_{s_{j}:s_{j+1}}^{\left(i\right)}\right)$

**Fig 1:**
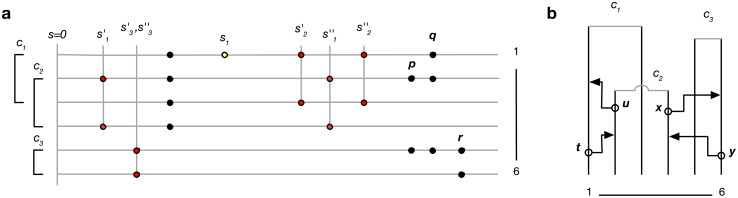
a. Example of data digest. Lines represent genomes of 6 lineages, circles observed genetic variants. Of the data shown, one singleton (yellow) and five doubletons (red) contribute to the digest. Cherry *c*_3_ is supported by a single doubleton; *r* does not contribute because the mutation patterns *p* and *q* are incompatible with *c*_3_. Similarly, *p* does not contribute because it is incompatible with *c*_2_ and *c*_3_. **b**. Partial genealogy (unbroken lines) over 6 lineages. Open circles and arrowheads represent potential recombination and coalescence events that would change the terminal branch length for lineage 1 (*t*,*u*), and remove cherry *c*_3_ (*x*,*y*).

   $v_{s_{j+1}}^{\left(i\right)}\leftarrow v_{s_{j}}^{\left(i\right)}\frac{\text{d}\pi_{x}}{\text{d}\xi_{x}}\left(x_{s_{j}:s_{j+1}}^{\left(i\right)}|X_{s_{j}}=x_{s_{j}}^{\left(i\right)}\right)\frac{\text{d}{\tilde{\pi }}^{s_{j+1}}}{\text{d}{\tilde{\pi }}^{s_{j}}}\left(y_{s_{j}:L}|X_{s_{j}:s_{j+1}}=x_{s_{j}:s_{j+1}}^{\left(i\right)}\right)$

(see Appendix, “Proof of Algorithm 3”.) To implement the lookahead particle filter we need a tractable approximate likelihood of future data given a current genealogy. To do this we simplify the full likelihood, and ignore all data except for a digest of singletons and doubletons that are informative of the topology and branch lengths near the tips of the genealogy—in particular, singletons are informative of terminal branch lengths, and doubletons identify the existence of nodes with precisely two descendants (“cherries”). This digest consists of the distance *s*_*i*_ to the nearest future singleton for each haploid sequence, and ≤ *n*/2 mutually consistent cherries *c*_*k*_ = (*a*_*k*_, *b*_*k*_) identified by their two descendants *a*_*k*_, *b*_*k*_, together with loci $s_{k}^{\prime }\leq s_{k}^{\prime \prime }$ where their first and last supporting doubleton were observed (Fig 1a). Under some simplifying assumptions we derive an approximation of the likelihood $h^{s}\left(\left\{t_{i}\right\},\left\{c_{k},s_{k}^{\prime },s_{k}^{\prime \prime }\right\}|x_{s}\right)$ of the current genealogy given these data; see Appendix (“A lookahead likelihood”) for details.

### Choosing waypoints

The choice of waypoints *s*_*j*_ can significantly impact the performance of the algorithm: choosing too few increases the variance of the approximation, and choosing too many slows down the algorithm without increasing its accuracy. Waypoints determine where the algorithms might perform a resampling step. A high density of waypoints is therefore always acceptable, but a low density may result in particle degeneracy. Choosing a waypoint at every event ensures that any weight variance induced at these sites is mitigated, but there is still the opportunity for weight variance to build up between events.

If *ξ*_*x*_ = *π*_*x*_, particle weights diverge only because different particles $\left(x_{1:s}^{\left(i\right)},w_{s}^{\left(i\right)}\right)$ experience a different total intensity $r(x_{s}^{\left(i\right)})$ of observed events. If *ESS*_0_ is the current estimated sample size, then under some assumptions, along an interval of length *L* where no events occur we have

$$\begin{array}{c} ESS\geq ESS_{0}e^{-\sigma^{2}L^{2}} \end{array}$$ (10)

(see Appendix, “Particle weight variance”), where *σ*^2^ the variance of the total event intensity *r*(*x*_*s*_) (9) under the prior *π*_*x*_(*x*). Therefore, if we choose waypoints at every event, adding additional waypoints so that they are never more than a distance $1/\sqrt{2\sigma^{2}}$ apart, the ESS will not drop more than a factor $\sqrt{1/e}\approx 0.6$ between waypoints, and particle degeneracy is avoided.

To apply this to our situation, assume a panmictic population with constant diploid effective population size *N*_*e*_. The variance of the total coalescent branch length in a sample of *n* individuals is ${\left(4N_{e}\right)}^{2}\sum_{i=1}^{n-1}i^{-2}$ [54]. The variance of total mutation intensity *σ*^2^ is obtained by multiplying this by *μ*^2^, since the rate of mutations on the coalescent tree is *μ* times the total branch length. Rewriting this in terms of the heterozygosity *θ* = 4*N*_*e*_ *μ*, and approximating the sum with $\sum_{i=1}^{\infty }i^{-2}=\pi^{2}/6$ gives *σ*^2^ = *θ*^2^ *π*^2^/6, and a minimum waypoint distance of $1/\sqrt{2\sigma^{2}}=\sqrt{3}/\pi \theta \approx 1/2\theta$.

Because the assumptions mentioned above are in practice only met approximately, this minimum waypoint density should be taken as a guide; breakdown of the assumptions can be monitored by tracking the *ESS*, increasing the density of waypoints if necessary.

### Parameter inference

Parameters can be inferred by stochastic expectation maximization (SEM), which involves maximizing the expected log likelihood over the posterior distribution of the latent variable. The probability density for a Poisson process is $\frac{1}{c!}\theta^{c}e^{-q\theta }$, where *c* is the event count, and *θ* is the rate of events per unit of “opportunity” *q*, measured in units of time or space or some combination of them. The expected log likelihood *c* log *θ* − *qθ* (ignoring constants) is maximized for *θ* = *c*/*q*, where *c* and *q* are the *expected* event count and opportunity. We consider Markov jump processes *X*_*s*_ with parameters *θ* and distribution

$$\begin{array}{c} \pi_{x}\left(x|\theta \right)=\prod_{i}\frac{1}{{\left|x\right|}_{i}!}\text{exp}\left\{-\theta_{i}Q_{i}\left(x\right)\right\}\theta_{i}^{{\left|x\right|}_{i}}\text{d}x, \end{array}$$ (11)

where |*x*|_*i*_ is the event count and *Q*_*i*_(*x*) is the total opportunity for events of type *i* in realisation *x*; both can be random variables. Similar to the Poisson case, the parameters maximizing the expected log likelihood are

$$\begin{array}{c} \theta_{i,\text{EM}}^{\prime }=\frac{E_{\pi (x|y,\theta )}{\left[\;|x\right|}_{i}\;]}{E_{\pi (x|y,\theta )}\left[\;Q_{i}\left(x\right)\;\right]} \end{array}$$ (12)

The expectations can be computed by using samples over *x* ∼ *π*(*x*|*y*, *θ*) as approximated by Algorithm 3.

To evaluate the expectations above we do not use the complete set of events in the full realisations *x*, since resampling causes early parts of *x* to become degenerate due to “coalescences” of the particle’s history of events along the sequence, which would lead to high variance of the estimates. Using only the most recent events is also problematic as these have not been shaped by many observations and mostly follow the prior *π*_*x*_(*x*|*θ*), resulting in highly biased estimates. Smoothing techniques such as two-filter smoothing [55] cannot be used here since finite-time transition probabilities are intractable. For discrete-time models fixed-lag smoothing is often effective [39]. For our model the optimal lag depends on the epoch, as the age of tree nodes strongly influence their correlation distance. For each epoch we determine the correlation distance empirically, and for the lag we use this distance multiplied by a factor *α*; we obtain good results with *α* = 1.

Particularly in cases where some event types are rare, Variational Bayes can improve on EM by iteratively estimating posterior distributions rather than point estimates of *θ*. A tractable algorithm is obtained if the joint posterior *π*(*x*, *θ*|*y*)d*x*d*θ* is approximated as a product of two independent distributions over *x* and *θ*, and an appropriate prior over *θ* is chosen. For the Poisson example above, combining a Γ(*θ*|*α*_0_, *β*_0_) prior with the likelihood *θ*^*c*^ *e*^−*qθ*^ results in a Γ(*θ*|*α*_0_ + *c*, *β*_0_ + *q*) posterior. Similarly, with this choice the Variational Bayes approximation results in an inferred posterior distribution of the form

$$\begin{array}{c} \theta_{i,\text{VB}}^{\prime }∼\Gamma (\alpha_{0}+{E[\;|x|}_{i}\;],\beta_{0}+E\left[\;Q_{i}\left(x\right)\;\right]) \end{array}$$ (13)

where expectations are taken over *x* ∼ ∫*π*(*x*|*y*, *θ*)*π*(*θ*)d*θ*, and *π*(*θ*) is the current posterior over *θ*. It would appear that obtaining samples *x* from this distribution is intractable. However, if *π*(*θ*) is a Gamma distribution, *θ* can be integrated out analytically in the likelihood *π*(*x*, *y*|*θ*)Γ(*θ*|*α*, *β*), resulting in an expression that is identical to the likelihood of the point estimate *θ*_*i*_ = *α*_*i*_/*β*_*i*_ except for an additional scaling factor *e*^*ψ*(*α*_*i*_)^/*α*_*i*_ for each event of type *i* in *x*, where *ψ* is the digamma function. These scaling factors render the normalization constant of the likelihood intractable, but fortunately SMC algorithms only require densities to be defined up to normalization. As a result, Algorithm 3 can be used to generate samples from this distribution at no additional computational cost. See the Appendix (“Variational Bayes for Markov Jump processes”) for more details.

Explicitly, for model (1) the parameters $\theta^{\prime }=\left(\rho_{\text{EM}},C_{\text{EM}}\right)$ maximising $E[\text{log}\;\pi_{x}\left(x|\theta^{\prime }\right)]$, where the expectation is taken over the posterior *x* ∼ *π*(*x*|*y*, *θ*)d*x* as approximated by Algorithm 3, is

$$\begin{array}{ccc} \rho_{\text{EM}}^{\prime } & =\frac{E[\;|x|\;]}{E[\int B\left(x_{s}\right)\text{d}s]}\;\;\text{and}\;\;C_{\text{EM}}^{\prime } & =\frac{E[\;|x|\;]}{E[\sum_{j=1}^{\left|x\right|}\int_{\nu_{j}}^{\tau_{j}}b_{u}\left(x_{s_{j}}\right)]}, \end{array}$$ (14)

where *θ* = (*ρ*, *C*) is the vector of current parameter estimates. Note that $C_{\text{EM}}^{\prime }$ in (14) is constant in evolutionary time. In practice we maximize (1) with respect to piecewise constant functions $C_{\text{EM}}^{\prime }\left(t\right)$, which yields

$$\begin{array}{c} C_{\text{EM}}^{\prime }\left(t\right)=\frac{E[\;|x{|}_{\nu ,\tau }\;]}{E[\sum_{j=1}^{\left|x\right|}\int_{u\in \left[\nu_{j},\tau_{j}\right)\cap \left[\nu ,\tau \right)}b_{u}\left(x_{s_{j}}\right)\text{d}u]} \end{array}$$ (15)

for *t* ∈ [*ν*, *τ*), where |*x*|_*ν*,*τ*_ denotes the number of coalescent events in *x* that occur in the epoch [*ν*, *τ*). Similarly, a Variational Bayes inference procedure uses

$$\begin{array}{c} \rho_{\text{VB}}^{\prime }∼\Gamma (\alpha_{\rho }+E\left[\;|x|\;\right],\beta_{\rho }+E\left[\int B\left(x_{s}\right)\text{d}s\right]) \end{array}$$ (16)

$$\begin{array}{c} C_{\text{VB}}^{\prime }∼\Gamma (\alpha_{C}+E\left[\;|x|\;\right],\beta_{C}+E\left[\sum_{j=1}^{\left|x\right|}\int_{\nu_{j}}^{\tau_{j}}b_{u}\left(x_{s_{j}}\right)\right]) \end{array}$$ (17)

where expectations are taken over *x* ∼ ∫*π*(*x*|*y*, *θ*)*p*(*θ*)d*θ*, where *p*(*θ*) is the posterior parameter distribution (16 and 17) of the previous iteration, and *α*_*ρ*_, *β*_*ρ*_, *α*_*C*_, *β*_*C*_ parameterize the prior distributions *ρ* ∼ Γ(*α*_*ρ*_, *β*_*ρ*_) and *C* ∼ Γ(*α*_*C*_, *β*_*C*_).

## Results

### Simulation study

We implemented the model and algorithm above in a Python/C++ program SMCSMC (Sequential Monte Carlo for the Sequentially Markovian Coalescent) and assessed it on simulated and real data.

**Fig 2:**
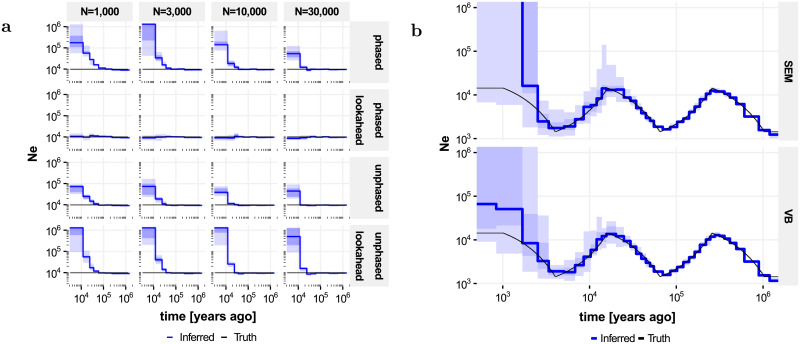
Accuracy of population size inferences in simulated data. Shown are true population sizes (black) and median inferred population sizes across 15 independent runs (blue); shaded areas denote quartiles and full extent. **a** Impact of lookahead, phasing and number of particles on the bias in population size estimates for recent epochs, for data simulated under a constant population size model. **b** Inference in the “zigzag” model on phased data using lookahead and 30, 000 particles, comparing inference using stochastic Expectation Maximization (SEM) and Variational Bayes (VB).

To investigate the effect of the lookahead particle filter, we simulated four 50 megabase (Mb) diploid genomes under a constant population-size model (*N*_*e*_ = 10, 000, *μ* = 2.5 × 10^−8^ and *ρ* = 10^−8^, both per generation and per site, generation time *g* = 30 years). We inferred population sizes *N*_*e*_ through evolutionary time, defined as the inverse of twice the instantaneous coalescent rate, as a piecewise constant function across 9 epochs (with boundaries at 400, 800, 1200, 2*k*, 4*k*, 8*k*, 20*k*, 40*k* and 60*k* generations) using particle filters Algorithms 2 and 3, as well as a recombination rate, which was taken to be constant through evolutionary time (and along the genome). Although recombination rate can be inferred, we here focus on the accuracy of the inferred *N*_*e*_ through evolutionary time. Observations are often available as unphased genotypes, and we assessed both algorithms using phased and unphased data, using the same simulations for both. Experiments were run for 15 EM iterations and repeated 15 times (Fig 2a).

On phased data (Fig 2a, top rows), *N*_*e*_ values inferred without lookahead show a strong positive bias in recent epochs, corresponding to a negative bias in the inferred coalescence rate. Increasing the number of particles reduces this bias somewhat. By contrast, the lookahead filter shows no discernable bias on these data, even for as little as 1, 000 particles. On unphased data (Fig 2a, bottom rows), the default particle filter continues to work reasonably well; in fact the bias appears somewhat reduced compared to phased data analyses, presumably because integrating over the phase makes the likelihood surface smoother, reducing particle degeneracy. By contrast, the lookahead particle filter shows an increased bias on these data compared to the default implementation. This is presumably because of the reliance of the lookahead likelihood on the distance to the next singleton; this statistic is much less informative for unphased data, making the lookahead procedure less effective, and even counterproductive for early epochs.

**Fig 3:**
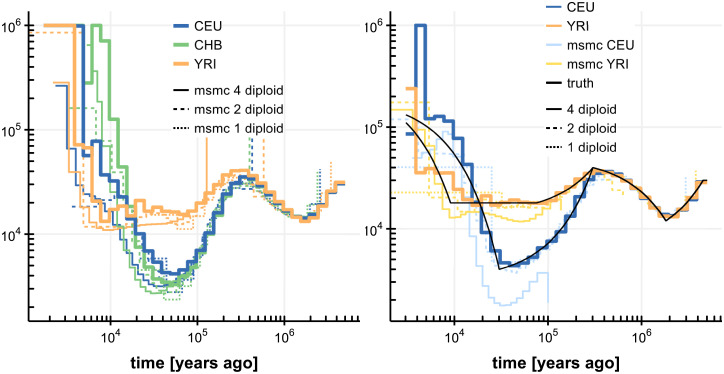
Population size inferences by SMCSMC on four diploid samples. Left, three human populations (CEU, CHB, YRI), together with inferences from msmc using 1, 2 and 4 diploid samples. Right, simulated populations resembling CEU and YRI population histories. All inferences (SMCSMC, msmc) were run for 20 iterations.

We next investigated the impact of using Variational Bayes instead of stochastic EM, using the lookahead filter on phased data. We simulated four 2 gigabase (Gb) diploid genomes using human-like evolutionary parameters (*μ* = 1.25 × 10^−8^, *ρ* = 3.5 × 10^−8^, *g* = 29, *N*_*e*_(0) = 14312) under a “zigzag” model similar to that used in [16] and [18], and inferred *N*_*e*_ across 37 approximately exponentially spaced epochs; see Appendix (“Implementation Details”). Both approaches give accurate *N*_*e*_ inferences from 2, 000 years up to 1 million years ago (Mya); other experiments indicate that population sizes can be inferred up to 10 Mya (but see Fig 3b). The upwards bias in the most recent epochs is reduced considerably by the Variational Bayes approach compared to SEM (Fig 2b), although some bias remains.

### Inference on human subpopulations

We applied SMCSMC to three sets of four phased diploid samples, of Northern European (CEU), Han Chinese (CHB) and Yoruban (YRI) ancestry respectively, from the 1000 Genomes project. For comparison we also ran msmc [16] inferring on the same data, and on subsets of 2 and 1 diploid samples. Inferences show good agreement where msmchas power (Fig 3). Since the inferences show some variation particularly in more recent epochs, we simulated data under a demographic model closely resembling CEU and YRI ancestry as inferred by multiple methods (see Appendix, “Implementation Details”), and we inferred population sizes using SMCSMC and msmc as before. This confirmed the accuracy of SMCSMC inferences from about 5,000 to 5 million years ago, while inferences in more recent epochs show more variability. A representative comparison of run times is provided in Table 1.

**Table 1:** Runtimes (total CPU time, hours) for analyzing one or two diploid human genomes using msmc (40 EM iterations), and SMCSMC (15 Variational Bayes iterations). Table lists means ± one standard deviation across 10 independent runs in a high performance compute environment. Note that due to parallel execution of SMCSMC (146 genomic chunks) and msmc (8 cores), wall clock time was considerably less than the total CPU time.

Algorithm	2 haploids	4 haploids
msmc	5.2±0.5	107.3±18.7
SMCSMC 5,000 particles	109.2±5.7	277±15
SMCSMC 10,000 particles	219±11	673±32

## Discussion

Motivated by the problem of recovering a population’s demographic history from the genomes of a sample of its individuals [1], we have introduced a continuous-locus approximation of the CwR model, and developed a particle filter algorithm for continuous-time Markov jump processes with a continuous phase space, by evaluating the doubly-continuous process at a suitably chosen set of “waypoints”, and applying a standard particle filter to the resulting discrete-time continuous-state process. It however proved very challenging to obtain reliable parameter inferences for our intended application using this approach. To overcome this challenge we have extended the standard particle filter algorithm in two ways. First, we have generalized the Auxiliary Particle Filter of Pitt and Shephard [40] from a discrete-time one-step-lookahead algorithm to a continuous-time unbounded-lookahead method. This helped to address a challenging feature of the CwR model, namely that recent demographic events induce “sticky” model states with very long forgetting times. With an appropriate lookahead likelihood function (and phased genotype data), we showed that the unbounded-lookahead algorithm mitigates the bias that is otherwise observed in the inferred parameters associated with these recent demographic events. Some bias however remained, particularly for very early epochs. We reduced this remaining bias by a Variational Bayes alternative to stochastic expectation maximization (SEM), which explicitly models part of the uncertainty in the inferred parameters, and avoid zero rate estimates which are fixed points for the SEM procedure. The combination of a continuous-time particle filter, the unbounded-lookahead method, and VB inference, allowed us to infer demographic parameters from up to four diploid genomes across many epochs, without making model approximations beyond passing to the continuous-locus limit.

On three sets of four diploid genomes, from individuals of central European, Han Chinese and Yoruban (Nigeria) ancestry respectively, we obtain inferences of effective population size over epochs ranging from 5,000 years to 5 million years ago. These inferences agree well with those made with other methods [14–18], and show higher precision across a wider range of epochs than was previously achievable by a single method. Despite the improvements from the unbounded-lookahead particle filter and the Variational Bayes inference procedure, the proposed method still struggles in very recent epochs (more recent than a few thousand years ago), and haplotype-based methods [e.g., 12] remain more suitable in this regime. In addition, methods focusing on recent demography benefit from the larger number of recent evolutionary event present in larger samples of individuals, and the proposed model will not scale well to such data, unless model approximations such as those proposed in [18] are used.

A key advantage of particle filters is that they are fundamentally simulation-based. This allowed us to perform inference under the full CwR model without having to resort to model approximations, such as requiring coalescences to occur at certain evolutionary times only, that characterizes most other approaches. The same approach will make it possible to analyze complex demographic models, as long as forward simulation (along the sequential variable) is tractable. The proposed particle filter is based on the sequential coalescent simulator SCRM [45], which already implements complex models of demography that include migration, population splits and mergers, and admixture events. Although not the focus of this paper, it should therefore be straightforward to infer the associated model parameters, including directional migration rates. In addition, several aspects of the standard CwR model are known to be unrealistic. For instance, gene conversions and doublet mutations are common [56, 57], and background selection profoundly shapes the heterozygosity in the human genome [58]. These features are absent from current models aimed at inferring demography, but impact patterns of heterozygosity and may well bias inferences of demography if not included in the model. As long as it is possible to include such features into a coalescent simulator, a particle filter can model such effects, reducing the biases otherwise expected in other parameter due to model misspecification. Because a particle filter produces an estimate of the likelihood, any improved model fit resulting from adding any of these features can in principle be quantified, if these likelihoods can be estimated with sufficiently small variance. However, even improved models will capture only a fraction of relevant features of a population’s evolution, and the inferred effective population sizes will continue to have a complex relationship with census population due to population substructure, variation in family size, and many other aspects [59].

A further advantage of a particle filter is that it provides a sample from the posterior distribution of ancestral recombination graphs (ARGs). Such explicit samples simplify the estimation of the age of mutations and recombinations, and explicit identification of sequence tracks with particular evolutionary histories, for instance tracts arising from admixture by a secondary population. In contrast to MCMC-based approaches [13], a particle filter can provide only one self-consistent sample of an ARG per run. However, for marginal statistics such as the expected age of a mutation or the expected number of recombinations in a sequence segment, a particle filter can provide weighted samples from the posterior in a single run.

The algorithm presented here scales in practice to about 4 diploid genomes, but requires increasingly large numbers of particles as larger numbers of genomes are analyzed jointly. This is because the space of possible tree topologies increases exponentially with the number of genomes observed, while the number of informative mutations grows much more slowly, resulting in increasing uncertainty in the topology given observed mutations. This uncertainty is further compounded by uncertainty in branch lengths. Nevertheless, the many effectively independent genealogies underlying even a single genome provide considerable information about past demographic events [14], and a joint analysis of even modest numbers of genomes under demographic models involving migration and admixture events enable more complex demographic scenarios to be investigated. Our results show that particle filters are a viable approach to demographic inference from whole-genome sequences, and the ability to handle complex model without having to resort to approximations opens possibilities for further model improvements, hopefully leading to more insight in our species’ recent demographic history.

## Appendix

### Conditional distributions and the Markov property

Here we outline how to define a conditional distribution π(·|G) given a distribution *π* on X and a conditioning subset G⊂X of measure 0. Suppose $G_{\tau }$ is a family of subsets of X so that $\cup_{\tau }G_{\tau }=X$. A particular subset $G_{\tau }$ for a fixed *τ* plays the role of the conditioning event *B* in the standard definition *P*(*A*|*B*) = *P*(*A* ∩ *B*)/*P*(*B*). It can be shown that, under some conditions, there exists an essentially unique family of measures $\pi_{G_{\tau }}$ and a measure *μ* so that $\pi_{G_{\tau }}$ is concentrated on $G_{\tau }$, $\pi_{G_{\tau }}\left(X\right)=1$ for all *τ*, and $E_{\pi }\left[f\right]=\;\int \int \;f\left(x\right)\pi_{G_{\tau }}\left(\text{d}x\right)\mu \left(\text{d}\tau \right)$ for well-behaved functions *f* [60], making it possible to define the conditional expectation as $E_{\pi }[f|G]=E_{\pi_{G}}[f]=\int f(x)\pi_{G}(\text{d}x).$ Using this, the Markov property of *π* can be expressed in terms of conditional expectations:

$$\begin{array}{c} E_{\pi }\left[f\left(x_{t}\right)|x_{s_{1}}=\tau_{1},\ldots ,x_{s_{k}}=\tau_{k}\right]=E_{\pi }\left[f\left(x_{t}\right)|x_{s_{k}}=\tau_{k}\right] \end{array}$$ (18)

for loci *s*_1_ < *s*_2_ < … < *s*_*k*_ < *t* and any well-behaved function *f*.

### Proof of Algorithm 3

The algorithm is proved by induction on *j*. For *j* = 0 the loop invariant holds, while for *j* = *K* it implies the output condition. Suppose the loop invariant is true for some *j*. If *ESS* < *N*/2, assume w.l.o.g. that $v^{\left(i\right)}=v_{s_{j}}^{\left(i\right)}$ are normalized, let *i*_*k*_ be the index of the *k*th new particle, ${\hat{v}}^{\left(k\right)}=N^{-1}$ and ${\hat{w}}^{\left(k\right)}=N^{-1}w^{\left(i_{k}\right)}/v^{\left(i_{k}\right)}$ be its weights, and write $\pi^{j}=\pi \left(X_{1:s_{j}}|Y_{1:s_{j}}=y_{1:s_{j}}\right)$, ${\tilde{\pi }}^{j}={\tilde{\pi }}^{s_{j}}\left(X_{1:s_{j}}|Y_{1:s_{j}}=y_{1:s_{j}}\right)$, then

$$\begin{array}{cc} & E_{\left\{\;v^{\left(i\right)}\;\right\}}\left[\sum_{k=1}^{N}{\hat{v}}^{\left(k\right)}f\left(X^{\left(i_{k}\right)}\right)\right]=\frac{1}{N}\;\sum_{k=1}^{N}E_{\left\{\;v^{\left(i\right)}\;\right\}}\left[f\left(X^{\left(i_{k}\right)}\right)\right]=\frac{1}{N}\;\sum_{k=1}^{N}\sum_{i=1}^{N}v^{\left(i\right)}f\left(X^{\left(i\right)}\right)\approx E_{{\tilde{\pi }}^{j}}\left[f\left(X\right)\right], \end{array}$$

and

$$\begin{array}{cc} & E_{\left\{\;v^{\left(i\right)}\;\right\}}\left[\sum_{k=1}^{N}{\hat{w}}^{\left(k\right)}f\left(X^{\left(i_{k}\right)}\right)\right]=\frac{1}{N}\;\sum_{k=1}^{N}E_{\left\{\;v^{\left(i\right)}\;\right\}}\left[\frac{w^{\left(i_{k}\right)}}{v^{\left(k\right)}}f\left(X^{\left(i_{k}\right)}\right)\right]=\frac{1}{N}\;\sum_{k=1}^{N}\sum_{i=1}^{N}w^{\left(i\right)}f\left(X^{\left(i\right)}\right)\approx E_{\pi^{j}}\left[f\left(X\right)\right], \end{array}$$

so that the loop invariant continues to hold after the optional resampling step.

After sampling $x_{s_{j}:s_{j+1}}^{\left(i\right)}∼\xi_{x}\left(X_{s_{j}:s_{j+1}}|X_{s_{j}}=x_{s_{j}}^{\left(i\right)}\right)$, the particles $\left\{\left(x_{1:s_{j}}^{\left(i\right)},w_{s_{j}}^{\left(i\right)}\right)\right\}$ approximate $\pi \left(X_{1:s_{j}}|Y=y_{1:s_{j}}\right)\xi_{x}\left(X_{s_{j}:s_{j+1}}|X_{s_{j}}\right)$. To make this distribution absolutely continuous w.r.t. $\pi (X_{1:s_{j+1}},Y_{s_{j}:s_{j+1}}|Y_{1:s_{j}})$, multiply it with the constant measure $\lambda_{s_{j}:s_{j+1}}\left(y_{s_{j}:s_{j+1}}\right)$; any measure will do as long as it has a density w.r.t. $\lambda_{s_{j}:s_{j+1}}$ and is independent of *X*. Taking the Radon-Nikodym derivative of these two distributions gives

$$\begin{array}{cc} & \frac{\text{d}\pi^{1:s_{j+1}}\left(X_{1:s+1},Y_{s_{j}:s_{j+1}}|Y_{1:s_{j}}\right)}{\text{d}[\pi^{1:s_{j}}\left(X_{1:s_{j}}|Y_{1:s_{j}}\right)\xi_{x}^{s_{j}:s_{j+1}}\left(X_{s_{j}:s_{j+1}}|X_{s_{j}}\right)\lambda^{s_{j}:s_{j+1}}\left(Y_{s_{j}:s_{j+1}}\right)]}\left(x_{1:s_{j+1}},y_{s_{j}:s_{j+1}}\right)\\ = & =\frac{\text{d}[\pi^{1:s_{j}}\left(X_{1:s_{j}}|Y_{1:s_{j}}\right)\pi_{x}^{s_{j}:s_{j+1}}\left(X_{s_{j}:s_{j+1}}|X_{s_{j}}\right)\pi^{s_{j}:s_{j+1}}\left(Y_{s_{j}:s_{j+1}}|X_{s_{j}:s_{j+1}}\right)]}{\text{d}[\pi^{1:s_{j}}\left(X_{1:s_{j}}|Y_{1:s_{j}}\right)\xi_{x}^{s_{j}:s_{j+1}}\left(X_{s_{j}:s_{j+1}}|X_{s_{j}}\right)\lambda^{s_{j}:s_{j+1}}\left(Y_{s_{j}:s_{j+1}}\right)]}\left(x_{1:s_{j+1}},y_{s_{j}:s_{j+1}}\right)\\ = & =\frac{\text{d}\pi_{x}}{\text{d}\xi_{x}}\left(x_{s_{j}:s_{j+1}}|X_{s_{j}}=x_{s_{j}}\right)\frac{\text{d}\pi }{\text{d}\lambda }\left(y_{s_{j}:s_{j+1}}|X_{s_{j}:s_{j+1}}=x_{s_{j}:s_{j+1}}\right) \end{array}$$

This shows that $\left\{w_{s_{j+1}}^{\left(i\right)},X_{1:s_{j+1}}^{\left(i\right)}\;\right\}$ form particles approximating $\pi^{1:s_{j+1}}\left(X_{1:s+1},y_{s_{j}:s_{j+1}}|y_{1:s_{j}}\right)$, and since $\pi \left(x_{1:s_{j+1}},y_{s_{j}:s_{j+1}}|Y_{1:s_{j}}\;=y_{1:s_{j}}\right)\;\propto \;\pi \left(x_{1:s_{j+1}}|Y_{1:s_{j+1}}\;=y_{1:s_{j+1}}\right)\lambda_{s_{j}:s_{j+1}}\left(y_{s_{j}:s_{j+1}}\right)$ they also approximate $\pi (x_{1:s_{j+1}}|Y_{1:s_{j+1}}=y_{1:s_{j+1}})$. The argument showing that $\left(v_{s_{j+1}}^{\left(i\right)},X_{1:s_{j+1}}^{\left(i\right)}\right)\approx {\tilde{\pi }}^{s_{j}}\left(x_{1:s_{j+1}}|Y\;=y_{1:L}\right)$ is analogous. This proves the loop invariant for *j* + 1, and the algorithm.

### Particle weight variance

To derive a criterion on the waypoints that limits the effect of weight variance build-up, let *R*(*s*) = *f*(*X*_*s*_) be the stochastic variable that measures the instantaneous rate of occurrence of emission events for a particular (random) particle *X*, and let $W\left(s\right)=W_{0}\text{exp}\left(-\;\int_{0}^{s}\;R\left(u\right)\text{d}u\right)$ be that particle’s time-dependent weight; the dependence on *W* on *X* is not written explicitly. Note that the expression for *W*(*s*) is valid as long as no events have occurred in the interval [0, *L*). We assume that *R*(*s*) is time-homogeneous, that it can be approximated by a Gaussian process, that particles are drawn from the equilibrium distribution, and that *W*_0_ and *R*(*s*) are independent. Write 〈*V*(*X*)〉 ≔ ∫*V*(*X*)*dπ*(*X*) for the expectation of *V* over *π*(*X*). Writing $R\left(s\right)=\mu +\tilde{R}\left(s\right)$ where *μ* = 〈*R*(*s*)〉 is the mean event rate (which is independent of *s* by assumption), then

$$\begin{array}{c} \left\langle W(L)\right\rangle =\left\langle W_{0}e^{-\int_{0}^{L}\mu +\tilde{R}\left(s\right)\text{d}s}\right\rangle =\left\langle W_{0}\right\rangle e^{-\mu L}\left\langle \prod_{i=1}^{k}\left(1-\tilde{R}\left(s_{i}\right)\Delta s\right)\right\rangle \end{array}$$ (19)

as *k* → ∞, where Δ*s* = *L*/*k* and *s*_*i*_ = *i*Δ*s*. The last expectation becomes

$$\begin{array}{cc} & \left\langle \sum_{n=0}^{\infty }{\left(-1\right)}^{n}\;\int_{0<s_{1}<\cdots <s_{n}<L}\;\tilde{R}\left(s_{1}\right)\cdots \tilde{R}\left(s_{n}\right)\text{d}s_{1}\cdots \text{d}s_{n}\right\rangle \\ = & \sum_{n=0}^{\infty }\frac{{\left(-1\right)}^{n}}{n!}\;\underset{s_{1},\ldots ,s_{n}=0}{\int^{L}}\;\left\langle \tilde{R}\left(s_{1}\right)\cdots \tilde{R}\left(s_{n}\right)\right\rangle \text{d}s_{1}\cdots \text{d}s_{n}\\ = & \sum_{m=0}^{\infty }\frac{1}{(2m)!}\frac{(2m-1)!}{2^{m-1}\left(m-1\right)!}{\left(\int_{s_{1},s_{2}=0}^{L}K\left(s_{1},s_{2}\right)\text{d}s_{1}\text{d}s_{2}\right)}^{m}\;=\sum_{m=0}^{\infty }\frac{1}{2^{m}m!}C^{m}=e^{\frac{1}{2}C} \end{array}$$

where in the second equality we used the formula for higher moments of a Gaussian distribution, *K* is the covariance function of the Gaussian process $\tilde{R}\left(t\right)$, and *C* is the integral $\int_{s_{1},s_{2}=0}^{L}K\left(s_{1},s_{2}\right)\text{d}s_{1}\text{d}s_{2}$. Now define *σ*^2^ ≔ *K*(*s*, *s*) and assume that the covariance function satisfies 0 ≤ *K*(*s*_1_, *s*_2_) ≤ *σ*^2^, then 0 ≤ *C* ≤ *σ*^2^ *L*^2^ and

$$\begin{array}{cc} \left\langle W\right\rangle & =\left\langle W_{0}\right\rangle e^{-\mu L+\frac{1}{2}C}\geq \left\langle W_{0}\right\rangle e^{-\mu L}, \end{array}$$ (20)

$$\begin{array}{cc} \left\langle W^{2}\right\rangle & =\left\langle W_{0}^{2}\right\rangle e^{-2\mu L+C}\leq \left\langle W_{0}^{2}\right\rangle e^{-2\mu L+\sigma^{2}L^{2}}, \end{array}$$ (21)

so that across an interval [0, *L*) where no events occur,

$$\begin{array}{c} ESS=\frac{{\left(\sum_{i=1}^{N}w^{\left(i\right)}\right)}^{2}}{\sum_{i=1}^{N}{\left(w^{\left(i\right)}\right)}^{2}}\approx \frac{N^{2}{\left\langle W\right\rangle }^{2}}{N\langle W^{2}\rangle }\geq \frac{N{\left\langle W_{0}\right\rangle }^{2}e^{-2\mu L}}{\left\langle W_{0}^{2}\right\rangle e^{-2\mu L+\sigma^{2}L^{2}}}=ESS_{0}e^{-\sigma^{2}L^{2}} \end{array}$$ (22)

where *ESS*_0_ is the expected sample size at *s* = 0, and ≈ denotes convergence in distribution as *N* → ∞ as before.

In practice particles will not be drawn from the equilibrium distribution *π*_*x*_(*X*), but from the joint distribution on *X* and *Y* conditioned on observations *y*. However, for most likelihoods conditioning will reduce the variance of *R* as observations tend to constrain the distribution of likely particles, making this a conservative assumption. The other assumption that is likely not met is that *R*(*t*) is a Gaussian process; it is less clear whether making this approximation will in practice be conservative.

### The sequential coalescent with recombination process

In formula (1), if *s* is a recombination point, *x*_*s*_ is the genealogy just *left* of the recombination point and includes the infinite branch from the root, so that *b*_*u*_(*x*_*s*_) = 1 for *u* above the root.

The measure (1) describes the CwR process exactly as long as *x* encodes both the local genealogy and the non-local branches used by the SCRM algorithm. In practice the SCRM algorithm prunes some of these branches, and we use (1) on the pruned *x*.

Note that we take the view that the realisation *x* encodes not only the sequence of genealogies *x*_*s*_ but also the number of recombinations |*x*| (some of which may not change the tree), their loci $s_{j}=s_{j}^{x}$, and the recombination and coalescence times $\nu_{j}^{x}$ and $\tau_{j}^{x}$. This information is also kept in the implementation of the algorithm, and is used to calculate the sufficient statistics required for inference of the coalescence and recombination rates.

### Variational Bayes for Markov jump processes

We consider hidden Markov models where the latent variable follows a Markov jump process over x∈X, that with respect to a suitable measure d*x*d*y* admits a probability density of the form

$$\begin{array}{c} \pi_{xy}\left(x,y|\theta \right)\text{d}x\text{d}y=\pi_{y}\left(y|x\right)\prod_{i}\text{exp}\{-\theta_{i}B_{i}\left(x\right)\}\theta_{i}^{{\left|x\right|}_{i}}\text{d}x\text{d}y. \end{array}$$ (23)

Here, |*x*|_*i*_ is the event count for events of type *i* in realisation *x*, and *B*_*i*_(*x*) is the total opportunity for events of that type in *x*. For example, in our case

$$\begin{array}{c} B_{U}^{R}\left(x\right)=\int_{s}\int_{u\in U}b_{u}\left(x_{s}\right)\text{d}u\text{d}s;\;B_{U}^{C}\left(x\right)=\sum_{j=1}^{\left|x\right|}\int_{u\in [\nu_{j},\tau_{j}]\cap U}b_{u}\left(x_{s_{j}}\right)\text{d}u, \end{array}$$ (24)

and ${\left|x\right|}_{U}^{R}=\#\left\{j:\nu_{j}\in U\right\}$, ${\left|x\right|}_{U}^{C}=\#\left\{j:\tau_{j}\in U\right\}$, for recombinations and coalescence opportunities and counts occurring in an epoch *U* ⊂ [0, ∞).

A Variational Bayes approach approximates the true joint posterior density *π*(*x*, *θ*|*y*) ∝ *π*_*xy*_(*x*, *y*|*θ*)*π*_*θ*_(*θ*), where *π*_*θ*_ is a prior on the parameters, with a probability density *ϕ*(*x*, *θ*) that is easier to work with (here the constant of proportionality implied by “∝” hides a constant density λ(*y*)). Following Hinton and van Camp [61] and MacKay [62], we choose to constrain *ϕ* by requiring it to factorize as *ϕ*(*x*, *θ*) = *ϕ*_*x*_(*x*)*ϕ*_*θ*_(*θ*), and we choose to optimize it by minimizing the Kullback-Leibler divergence *KL*(*ϕ*||*π*), also referred to as the variational free energy [63],

$$\begin{array}{c} F\left(\phi \right)=-\int_{x}\int_{\theta }\phi \left(x,\theta \right)\text{log}[\frac{\pi (x,\theta |y)}{\phi (x,\theta )}]\text{d}\theta \text{d}x. \end{array}$$ (25)

To optimize *ϕ*_*θ*_(*θ*) we write *F*(*ϕ*) as a function of *ϕ*_*θ*_ with *ϕ*_*x*_ fixed, as

$$\begin{array}{cc} F(\phi ) & =-\;\int \;\phi_{x}\left(x\right)\phi_{\theta }\left(\theta \right)\{\sum_{i}{\left|x\right|}_{i}\text{log}\;\theta_{i}\;-\;B_{i}\left(x\right)\theta_{i}\;+\;\text{log}\;\pi_{\theta }\left(\theta \right)\;-\;\text{log}\;\phi_{\theta }\left(\theta \right)\}\text{d}\theta \text{d}x+const \end{array}$$ (26)

$$\begin{array}{cc} & =\int \;\phi_{\theta }\left(\theta \right)\text{log}\frac{\phi_{\theta }\left(\theta \right)}{\pi_{\theta }\left(\theta \right)\prod_{i}\theta_{i}^{E_{\phi_{x}}{\left[|x\right|}_{i}]}\text{exp}\left\{-E_{\phi_{x}}\left[B_{i}\left(x\right)\right]\theta_{i}\right\}}\text{d}\theta +const \end{array}$$ (27)

This is minimized by setting log*ϕ*_*θ*_(*θ*) equal to the log of the denominator. We can still choose the prior *π*_*θ*_(*θ*); a product of Gamma distributions $\prod_{i}\Gamma \left(\alpha_{i},\beta_{i}\right)\left(\theta_{i}\right)\propto \prod_{i}\theta_{i}^{\alpha_{i}-1}\text{exp}\left\{-\beta_{i}\theta_{i}\right\}$ is suitable as it is conjugate to the factors appearing in the denominator. The result is that

$$\begin{array}{c} \phi_{\theta }\left(\theta \right)=\prod_{i}\Gamma \left(\alpha_{i}^{\prime },\beta_{i}^{\prime }\right) \end{array}$$ (28)

with $\alpha_{i}^{\prime }=\alpha_{i}+E_{\phi_{x}}{\left[|x\right|}_{i}]$ and $\beta_{i}^{\prime }=\beta_{i}+E_{\phi_{x}}\left[B_{i}\left(x\right)\right]$. Next, to optimize *ϕ*_*x*_(*x*) we write *F*(*ϕ*) as a function of *ϕ*_*x*_ with *ϕ*_*θ*_ fixed,

$$\begin{array}{cc} F(\phi ) & =-\int \phi_{x}\left(x\right)\phi_{\theta }\left(\theta \right)\{\sum_{i}{\left|x\right|}_{i}\text{log}\;\theta_{i}-B_{i}\left(x\right)\theta_{i}+\text{log}\;\pi_{y}\left(y|x\right)-\text{log}\phi_{x}\left(x\right)\}\text{d}\theta \text{d}x+const\\ & =\int \phi_{x}\left(x\right)\text{log}\;\frac{\phi_{x}\left(x\right)}{\pi_{y}\left(y|x\right)\prod_{i}{\text{exp}\{|x|}_{i}E_{\phi_{\theta }}\left[\text{log}\;\theta_{i}\right]-B_{i}\left(x\right)E_{\phi_{\theta }}\left[\theta_{i}\right]\}}\text{d}x+const \end{array}$$

Define ${\bar{\theta }}_{i}:=E_{\phi_{\theta }}\left[\theta_{i}\right]$ and $\theta_{i}^{*}:=\text{exp}\left\{E_{\phi_{\theta }}\left[\text{log}\theta_{i}\right]\right\}$, then using properties of the Gamma distribution we get $\bar{\theta }=\alpha_{i}^{\prime }/\beta_{i}^{\prime }$ and $\theta_{i}^{*}=\text{exp}\left\{\psi \left(\alpha_{i}^{\prime }\right)-\text{log}\;\beta_{i}^{\prime }\right\}$ where *ψ* is the digamma function. Again, *F*(*ϕ*) is minimized if the numerator and denominator are proportional, which happens for

$$\begin{array}{cc} \phi_{x}\left(x\right) & \propto \pi_{y}\left(y|x\right)\;\prod_{i}\;\text{exp}\;\{-{\bar{\theta }}_{i}B_{i}\left(x\right)\}\;{\left(\theta_{i}^{*}\right)}^{{\left|x\right|}_{i}}\propto \pi \left(x|y,\bar{\theta }\right)\;\prod_{i}\;{\left(\frac{\theta_{i}^{*}}{{\bar{\theta }}_{i}}\right)}^{{\;|x|}_{i}}\;=\pi \left(x|y,\bar{\theta }\right)\;\prod_{i}\;\eta_{i}^{{\left|x\right|}_{i}} \end{array}$$ (29)

where $\eta_{i}:=\theta_{i}^{*}/{\bar{\theta }}_{i}=\text{exp}\left\{\psi \left(\alpha_{i}^{\prime }\right)\right\}/\alpha_{i}^{\prime }$. As given, the algorithms in this paper sample from a distribution of the form $\pi (x|y,\bar{\theta })$, but they can easily be modified to sample from *ϕ*_*x*_(*x*) instead by including an additional factor *η*_*i*_ in a particle’s weight for every event of type *i* that occurs.

### A lookahead likelihood

Let *s*_*i*_ denote the distance along the genome to the nearest future singleton in each sequence, and let *c*_*k*_ = (*a*_*k*_, *b*_*k*_) be ≤ *n*/2 mutually consistent cherries with loci $s_{k}^{\prime }\leq s_{k}^{\prime \prime }$ of their first and last supporting doubleton. To simplify notation we assume that the current locus is 0 (Fig 1a).

Note that recombinations result in a change of a terminal branch length (TBL) if either the recombination occurred in the branch itself and the new lineage does not coalesce back into it, or the recombination occurred outside the branch and the new lineage coalesces into it (Fig 1b). To compute the likelihood that the first singleton in lineage *i* occurs at locus *s*_*i*_, we assume that all TBLs are equal to *l*_*i*_, and that coalescences occur before *l*_*i*_. Then, the total rate of events that change the TBL *i* is

$$\begin{array}{c} \rho_{i}:=l_{i}\rho \frac{n-1}{n}+\left(n-1\right)l_{i}\rho \frac{1}{n}=2l_{i}\rho \frac{n-1}{n}. \end{array}$$

Define *μ*_*i*_ ≔ *μl*_*i*_ to be the total mutation rate on branch *i*, and assume that when a TBL changes, it reverts deterministically to some length $l_{i}^{\prime }$. If a terminal branch with length *l*_*i*_ changes at *u* to $l_{i}^{\prime }$, which happens with probability *e*^−*ρ*_*i*_*u*^ *ρ*_*i*_d*u*, the likelihood that the first singleton occurs at distance *s*_*i*_ is $e^{-\mu_{i}u}e^{-\mu_{i}^{\prime }\left(s_{i}-u\right)}\mu_{i}^{\prime }\text{d}t,$ where $\mu_{i}^{\prime }:=\mu l_{i}^{\prime }$. Conversely, if that branch does not change along [0, *s*_*i*_), which happens with probability $e^{-\rho_{i}s_{i}}$, the likelihood is $e^{-\mu_{i}s_{i}}\mu_{i}\text{d}t$. Combining these possibilities and marginalizing over *u* ∈ [0, *s*_*i*_) gives (using Mathematica to evaluate the integral)

$$\begin{array}{c} p\left(\text{1st}\;\text{singleton}\;\text{in}\;i\;\text{at}\;s_{i}|l_{i},l_{i}^{\prime }\right)=\frac{1}{\rho_{i}+\mu_{i}-\mu_{i}^{\prime }}(\rho_{i}\mu_{i}^{\prime }e^{-\mu_{i}^{\prime }s_{i}}+\left(\mu_{i}-\mu_{i}^{\prime }\right)\left(\rho_{i}+\mu_{i}\right)e^{-\left(\rho_{i}+\mu_{i}\right)s_{i}})\text{d}t. \end{array}$$

In the case that no singleton is observed up until *s*_*i*_ but data was missing thereafter, the same probability densities apply except for the factors *μ*_*i*_ and $\mu_{i}^{\prime }$ in the likelihood, so that

$$\begin{array}{c} p\left(\text{no}\;\text{singleton}\;\text{in}\;i\;\text{until}\;s_{i}|l_{i},l_{i}^{\prime }\right)=\frac{1}{\rho_{i}+\mu_{i}-\mu_{i}^{\prime }}(\rho_{i}e^{-\mu_{i}^{\prime }s_{i}}+\left(\mu_{i}-\mu_{i}^{\prime }\right)e^{-\left(\rho_{i}+\mu_{i}\right)s_{i}})\text{d}t. \end{array}$$ (38)

We account for the uncertainty in $l_{i}^{\prime }$ by marginalizing over the empirical distribution of TBLs for sequence *i*.

To approximate the likelihood of the doubleton data, note that a node *c* with precisely two descendants (*a*, *b*) (a “cherry”) at height *l* changes if a recombination occurs in either branch *a* or *b* and the new lineage coalesces out, or a recombination occurs outside of *a* and *b* and coalesces into either (Fig 1b). Again assuming that all TBLs are *l* and coalescences occur before *l*, the total rate of change is $2l\rho \frac{n-2}{n}+\left(n-2\right)l\rho \frac{2}{n}=4l\rho \frac{n-2}{n}:=\rho_{C}$. When a cherry changes, we assume that the new cherry is drawn from the equilibrium distribution. To calculate the probablity of observing *c* = (*a*, *b*) at equilibrium, assume that a tree supports 1 ≤ *k* ≤ *n*/2 cherries. The branches of *c* are among the 2*k* branches subtended by the tree’s *k* cherries with probability $\frac{2k}{n}\frac{2k-1}{n-1}$, and *a* is paired with *b* with probability $\frac{1}{2k-1}$. Since *k* has mean *n*/3 if *n* ≥ 3 [64], the probability of observing (*a*, *b*) at equilibrium is $\frac{2}{3(n-1)}$. We approximate the likelihood of a doubleton by 0 if the *c* is not in the tree, and by 1 if it is. Then, the likelihood of observing *c*_*k*_ = (*a*_*k*_, *b*_*k*_) at the last known locus $s_{k}^{\prime \prime }$ conditional on the tree currently containing *c*_*k*_ is

$$\begin{array}{c} p\left(a_{k},b_{k},s_{k}^{\prime },s_{k}^{\prime \prime }|\left(a_{k},b_{k};l\right)\in \tau \right)=e^{-\rho_{C}s_{k}^{\prime \prime }}+\frac{2}{3(n-1)}\left(1-e^{-\rho_{C}s_{k}^{\prime \prime }}\right), \end{array}$$ (30)

where (*a*_*k*_, *b*_*k*_;*l*) ∈ *τ* expresses that *τ* contains cherry *c*_*k*_ = (*a*_*k*_, *b*_*k*_) at height *l*. Now suppose *c*_*k*_ ∉ *τ* and let $\bar{l}$ be the average TBL in *τ*. Under similar assumptions, cherries are created at a rate $\left(n-1\right)\rho \bar{l}$ and assuming that new cherries are drawn from the equilibrium distribution, the likelihood of observing *c*_*k*_ at the first known locus $s_{k}^{\prime }$ is

$$\begin{array}{c} p\left(a_{k},b_{k},s_{k}^{\prime },s_{k}^{\prime \prime }|\bar{l},\left(a_{k},b_{k}\right)\notin \tau \right)=\frac{2}{3(n-1)}\left(1-e^{-\rho_{C}^{\prime }s_{k}^{\prime }}\right), \end{array}$$ (31)

where $\rho_{C}^{\prime }=\left(n-1\right)\bar{l}\rho$ is the effective rate of recombinations that potentially result in the creation of *c*_*k*_. Note that (30 and 31) are likelihoods for *τ* supporting *c*_*k*_ at the given locus, rather than for a doubleton mutation actually occurring.

These likelihoods show good performance, but result in some negative bias in inferred population size for recent epochs. We traced this to the lack of correlation between *l*_*i*_ and $l_{i}^{\prime }$, requiring a single very recent coalescence to explain a long segment devoid of singletons, rather than allowing for the possibility of several correlated coalescences each in slightly earlier epochs. To model correlations, we averaged the likelihood above over *ρ*′ = *ρ* and *ρ*′ = *ρ*/2 each weighted with probability 1/2. This effectively removed the negative bias.

To deal with missing data, we reduce *μ* proportionally to the missing segment length and the number of lineages missing. For unphased mutation data, singletons and doubletons can still be extracted, and are greedily assigned to compatible lineages. The likelihoods are also similarly calculated, by greedily assigning cherries to observed doubletons. Unphased singletons can result from mutations on either of the individual’s alleles; the likelihood term uses the sum of the two branch lengths for that individual to calculate the expected rate of unphased singletons.

### Implementation details

While *x*_1:*s*_ refers to the entire sequence of genealogies along the sequence segment 1: *s*, storing this sequence would require too much memory. Instead we only store the most recent genealogy *x*_*s*_ (including non-local branches where appropriate), which is sufficient to simulate subsequent genealogies using the SCRM algorithm. To implement epoch-dependent lags when harvesting sufficient statistics, we do store records of the events (recombinations, coalescences and migrations) that changed *x* along the sequence, as well as the associated opportunities, for each particle and each epoch; this implicitly stores the full ARG. To avoid making copies of potentially many event records when particles are duplicated at resampling, these are stored in a linked list, and are shared by duplicated particles where appropriate, forming a tree structure. Records are removed dynamically after contributing to the summary statistics, and when particles fail to be resampled, ensuring that memory usage is bounded.

The likelihood calculations involve many evaluations of the exponential function, often for small exponents. We use the continued-fraction approximation $e^{x}\approx 1+2x/\left(2-x+\frac{1}{6}x^{2}\right)$ for |*x*| < 0.03, with relative error bounded by 10^−10^ [65].

Table 2 shows the commands to generate the data for the three simulation experiments. Epoch boundaries for *N*_*e*_ inference in generations for the zigzag experiment were defined by taking interval boundaries −14312log(1−*i*/256)/2, *i* = 0, …, 255, merging intervals according to the pattern 4 * 1 + 7 * 2 + 8 * 5 + 7 * 13 + 1 * 15 + 8 * 11 + 1 * 3 (37 epochs; see [14]). For the real data experiments, epochs boundaries for the 32 epochs were logarithmically spaced from 133 to 133016 generations ago, using generation time *g* = 29 years, without merging intervals (command line option -P 133 133016 31*1).

**Table 2:** Commands to generate simulation data.

Experiment	Command
zigzag	scrm 8 1 -N0 14312 -t 1431200 -r 400736 2000000000 -eN 0 1 -eG 0.000582262 1318.18 -eG 0.00232905 -329.546 -eG 0.00931619 82.3865 -eG 0.0372648 -20.5966 -eG 0.149059 5.14916 -eN 0.596236 0.1 -seed 1 -T -L -p 10 -l 300000
CEU	scrm 8 1 -N0 14312 -t 1789000 -r 500920 2500000000 -eN 0 10.4807 -eG 0.00120468 214.8965 -eG 0.0180702 -14.15827 -eG 0.180702 1.33255 -eG 1.084212 -0.563414 -eN 2.71053 2.096143 -seed 1 -T -L -p 10 -l 300000
YRI	scrm 8 1 -N0 14312 -t 1789000 -r 500920 2500000000 -eN 0 10.4807 -eG 0.00120468 502.8635 -eG 0.00542106 0 -eG 0.0451755 -5.89189 -eG 0.180702 1.33255 -eG 1.084212 -0.563414 -eN 2.71053 2.096143 -seed 1 -T -L -p 10 -l 300000
